# Supplementary material for: Preventive effects of Salvia officinalis leaf extract on insulin resistance and inflammation in a model of high fat diet-induced obesity in mice that responds to rosiglitazone
Source: PeerJ. 2018 Jan 9;6:e4166. doi: 10.7717/peerj.4166 (PMC5765810; doi:10.7717/peerj.4166)
Supplement: Data S1 [file peerj-06-4166-s001.docx]

**Raw Data for Figure1**

**Figure 1A**

| 0.0 | 0.2 | 1.0 | 5.0 | 25 | 50 |
| --- | --- | --- | --- | --- | --- |
| 417.311400 | 378.822800 | 334.154500 | 384.255400 | 381.538800 | 390.596300 |
| 302.105300 | 352.390900 | 356.532000 | 315.967800 | 313.619800 | 290.940900 |
| 338.768500 | 341.469300 | 324.836800 | 348.874800 | 325.021400 | 368.951100 |
| 296.685800 | 369.547100 | 363.454300 | 305.680400 | 314.681700 | 372.592100 |

**Figure 1B**

| 0.0 | 0.2 | 1.0 | 5.0 | 25 | 50 |
| --- | --- | --- | --- | --- | --- |
| 0.495125 | 0.383125 | 0.466125 | 0.496125 | 0.357125 | 0.415125 |
| 0.519125 | 0.385125 | 0.469125 | 0.500125 | 0.356125 | 0.416125 |
| 0.497125 | 0.458125 | 0.392125 | 0.389125 | 0.456125 | 0.376125 |
| 0.495125 | 0.457125 | 0.383125 | 0.389125 | 0.455125 | 0.373125 |
| 0.439125 | 0.470125 | 0.422125 | 0.389125 | 0.389125 | 0.443125 |
| 0.438125 | 0.471125 | 0.422125 | 0.388125 | 0.388125 | 0.442125 |
| 0.388125 | 0.370125 | 0.393125 | 0.387125 | 0.340125 | 0.359125 |
| 0.386125 | 0.370125 | 0.393125 | 0.386125 | 0.337125 | 0.356125 |

**Figure 1C**

| 0.0 | 0.2 | 1.0 | 5.0 | 25 | 50 |
| --- | --- | --- | --- | --- | --- |
| 0.369125 | 0.369125 | 0.385125 | 0.398125 | 0.381125 | 0.374125 |
| 0.374125 | 0.374125 | 0.389125 | 0.399125 | 0.381125 | 0.381125 |
| 0.451125 | 0.385125 | 0.368125 | 0.398125 | 0.342125 | 0.343125 |
| 0.454125 | 0.386125 | 0.368125 | 0.398125 | 0.341125 | 0.340125 |
| 0.357125 | 0.381125 | 0.386125 | 0.322125 | 0.363125 | 0.332125 |
| 0.355125 | 0.380125 | 0.386125 | 0.323125 | 0.364125 | 0.332125 |
| 0.469125 | 0.439125 | 0.379125 | 0.414125 | 0.393125 | 0.342125 |
| 0.466125 | 0.433125 | 0.384125 | 0.413125 | 0.392125 | 0.338125 |

**Figure 1D**

| 0.0 | 0.2 | 1.0 | 5.0 | 25 | 50 |
| --- | --- | --- | --- | --- | --- |
| 0.435000 | 0.354000 | 0.339000 | 0.369000 | 0.312000 | 0.312000 |
| 0.433000 | 0.360000 | 0.344000 | 0.374000 | 0.313000 | 0.318000 |
| 0.351000 | 0.383000 | 0.380000 | 0.355000 | 0.285000 | 0.267000 |
| 0.351000 | 0.384000 | 0.381000 | 0.355000 | 0.286000 | 0.262000 |
| 0.383000 | 0.366000 | 0.374000 | 0.287000 | 0.341000 | 0.335000 |
| 0.386000 | 0.363000 | 0.376000 | 0.285000 | 0.342000 | 0.333000 |
| 0.429000 | 0.332000 | 0.345000 | 0.306000 | 0.334000 | 0.319000 |
| 0.429000 | 0.329000 | 0.343000 | 0.300000 | 0.332000 | 0.316000 |

**Raw Data for Figure2**

**Figure 2A**

| Time (min) | vehicle | | | | | | | | |
| --- | --- | --- | --- | --- | --- | --- | --- | --- | --- |
| -30. |  | 6.817 | 6.235 | 7.072 | 8.811 | 7.388 | 7.044 | 6.504 |  |
| 0. |  | 7.113 | 6.915 | 6.921 | 9.095 | 7.354 | 9.591 | 6.996 |  |
| 30. |  | 13.917 | 11.098 | 10.514 | 15.207 | 15.001 | 16.595 | 11.660 |  |
| 60. |  | 13.642 | 9.855 | 9.670 | 11.116 | 15.065 | 10.754 | 11.076 |  |
| 120. |  | 11.998 | 9.250 | 7.244 | 10.896 | 11.715 | 10.615 | 8.001 |  |
| 180. |  | 8.993 | 9.135 | 7.437 | 9.847 | 9.600 | 9.515 | 7.454 |  |

| Time (min) | Sage (100 mg/kg/day.bid) | | | | | | | | |
| --- | --- | --- | --- | --- | --- | --- | --- | --- | --- |
| -30. | 5.707 | 6.282 | 6.490 | 7.054 | 7.827 | 7.638 | 6.376 | 7.185 |  |
| 0. | 6.764 | 6.168 | 7.083 | 6.769 | 7.112 | 6.838 | 6.669 | 7.439 |  |
| 30. | 8.371 | 9.865 | 9.019 | 9.616 | 9.281 | 9.902 | 12.437 | 11.360 |  |
| 60. | 11.646 | 11.215 | 9.840 | 9.886 | 10.170 | 8.834 | 9.942 | 10.033 |  |
| 120. | 8.397 | 8.491 | 11.582 | 9.567 | 10.449 | 6.831 | 8.297 | 7.810 |  |
| 180. | 6.798 | 6.939 | 8.393 | 7.097 | 9.008 | 6.511 | 6.554 | 8.309 |  |

| Time (min) | Sage (400 mg/kg/day.bid) | | | | | | | |
| --- | --- | --- | --- | --- | --- | --- | --- | --- |
| -30. | 8.945 | 6.969 | 7.757 | 8.740 | 6.192 | 7.002 | 6.960 | 7.127 |
| 0. | 9.641 | 6.753 | 7.391 | 8.431 | 7.577 | 8.398 | 9.085 | 9.240 |
| 30. | 12.972 | 9.010 | 11.872 | 11.479 | 10.614 | 12.601 | 12.250 | 10.831 |
| 60. | 10.700 | 8.834 | 8.000 | 8.390 | 8.858 | 12.074 | 11.217 | 10.274 |
| 120. | 9.388 | 7.366 | 7.717 | 8.642 | 8.875 | 9.242 | 11.509 | 9.900 |
| 180. | 9.782 | 6.383 | 7.471 | 7.900 | 7.325 | 9.913 | 8.835 | 8.849 |

| Time (min) | Rosi.(3mg/kg/day.bid) | | | | | | | |
| --- | --- | --- | --- | --- | --- | --- | --- | --- |
| -30. | 5.975 | 5.440 | 7.569 | 6.981 | 5.536 | 7.119 | 6.412 | 7.074 |
| 0. | 6.401 | 7.053 | 6.599 | 6.000 | 6.487 | 7.255 | 7.590 | 6.915 |
| 30. | 9.585 | 9.363 | 9.354 | 10.770 | 11.235 | 9.837 | 11.021 | 12.609 |
| 60. | 10.550 | 9.590 | 9.532 | 9.555 | 10.468 | 9.570 | 11.551 | 10.636 |
| 120. | 7.494 | 7.873 | 8.078 | 7.166 | 7.325 | 9.390 | 10.404 | 9.212 |
| 180. | 7.161 | 7.232 | 8.299 | 7.328 | 6.754 | 7.263 | 7.815 | 8.102 |

**Figure 2B**

|  |  |  |  |  |  |  |  |  |  |
| --- | --- | --- | --- | --- | --- | --- | --- | --- | --- |
|  | vehicle | | | | | | | | |
| T = -30 |  | 908.14 | 351.47 | 522.95 | 1488.96 | 2128.51 | 1031.39 | 443.91 |  |
| T= +30 |  | 3851.50 | 1615.40 | 1148.93 | 3736.34 | 5131.62 | 4583.10 | 1146.27 |  |
|  |  |  |  |  |  |  |  |  |  |
|  | Sage (100 mg/kg/day.bid) | | | | | | | |  |
| T = -30 | 362.91 | 339.52 | 536.52 | 492.62 | 740.18 | 740.99 | 423.10 | 715.97 |  |
| T= +30 | 913.36 | 683.71 | 1155.01 | 1210.11 | 954.80 | 2648.74 | 1690.17 | 1904.86 |  |
|  |  |  |  |  |  |  |  |  |  |
|  | Sage (400 mg/kg/day.bid) | | | | | | | |  |
| T = -30 | 1746.24 | 386.24 | 649.30 | 926.39 | 475.85 | 916.48 | 1108.35 | 438.29 |  |
| T= +30 | 4177.56 | 633.23 | 1994.36 | 2531.43 | 1171.75 | 2135.07 | 2932.16 | 933.40 |  |
|  |  |  |  |  |  |  |  |  |  |
|  | Rosi.(3mg/kg/day.bid) | | | | | | | |  |
| T = -30 | 298.05 | 381.92 | 379.39 | 191.10 | 194.56 | 396.66 | 407.87 | 340.58 |  |
| T= +30 | 664.92 | 865.26 | 797.72 | 786.16 | 387.42 | 513.43 | 1243.84 | 1089.25 |  |

**Raw Data for Figure 3**

**Figure 3A**

| vehicle | Sage (100 mg/kg/day.bid) | Sage (400 mg/kg/day.bid) | Rosi.(3mg/kg/day.bid) |
| --- | --- | --- | --- |
|  | 6.5500 | 7.5000 | 6.2910 |
| 8.6410 | 6.7070 | 8.0870 | 7.2170 |
| 6.7990 | 7.6520 | 8.7600 | 7.6420 |
| 7.5460 | 8.4790 | 8.2380 | 7.5180 |
| 7.6520 | 7.2450 | 6.7520 | 6.4840 |
| 7.6310 | 7.5050 | 7.1540 | 7.0040 |
| 7.6100 | 7.1500 | 8.8060 | 7.7400 |
| 8.2460 | 8.1750 | 8.9310 | 7.5860 |

**Figure 3B**

| vehicle | | | Sage (100 mg/kg/day.bid) | | | | Sage (400 mg/kg/day.bid) | | | | Rosi.(3mg/kg/day.bid) | | | |
| --- | --- | --- | --- | --- | --- | --- | --- | --- | --- | --- | --- | --- | --- | --- |
|  | | | 2583.9420 | | | | 10996.4400 | | | | 1468.6860 | | | |
| 17713.7000 | | | 2708.5660 | | | | 4400.8780 | | | | 2661.0040 | | | |
| 5700.4860 | | | 4306.3030 | | | | 5135.9150 | | | | 2803.1870 | | | |
| 6148.1410 | | | 4114.8310 | | | | 5013.1530 | | | | 1342.8900 | | | |
| 10945.1900 | | | 5143.2860 | | | | 2650.7000 | | | | 841.4935 | | | |
| 13943.9500 | | | 3370.4330 | | | | 3582.9380 | | | | 1662.3060 | | | |
| 6682.7340 | | | 2002.5480 | | | | 9332.5110 | | | | 3198.6710 | | | |
| 3455.8020 | | | 5222.3800 | | | | 3832.7390 | | | | 2627.5880 | | | |
| **Figure 3C** | | |  |  |  | |  |  |  | |  |  |  |  |
| Time (min) | vehicle | | | | | | | | | | | |  |  |
| -10 |  | | | 7.769 | 7.164 | | 5.299 | 6.009 | 6.04 | | 6.249 | 7.319 |  |  |
| 0 |  | | | 9.967 | 9.629 | | 7.624 | 9.303 | 8.303 | | 9.566 | 6.467 |  |  |
| 10 |  | | | 10.281 | 9.169 | | 5.972 | 9.294 | 11.828 | | 9.641 | 7.785 |  |  |
| 20 |  | | | 9.572 | 6.182 | | 4.078 | 8.271 | 7.767 | | 8.442 | 7.729 |  |  |
| 30 |  | | | 7.702 | 6.154 | | 4.033 | 6.626 | 8.787 | | 8.559 | 4.272 |  |  |
| 45 |  | | | 6.681 | 4.754 | | 4.568 | 6.088 | 7.687 | | 7.651 | 4.412 |  |  |
| 60 |  | | | 6.691 | 5.36 | | 3.998 | 7.197 | 8.312 | | 6.875 | 4.27 |  |  |
| Time (min) | Sage (100 mg/kg/day.bid) | | | | | | | | | | | |  |  |
| -10 | 5.38 | | | 5.25 | 6.041 | | 6.179 | 6.397 | 5.188 | | 4.995 | 6.267 |  |  |
| 0 | 6.968 | | | 6.129 | 7.613 | | 7.741 | 8.843 | 7.016 | | 6.665 | 6.952 |  |  |
| 10 | 7.88 | | | 5.6 | 7.555 | | 6.695 | 8.5 | 7.361 | | 8.075 | 7.631 |  |  |
| 20 | 5.157 | | | 3.847 | 5.706 | | 5.312 | 6.147 | 6.012 | | 6.216 | 5.814 |  |  |
| 30 | 5.066 | | | 3.653 | 5.252 | | 5.628 | 6.854 | 5.798 | | 4.681 | 6.598 |  |  |
| 45 | 4.718 | | | 4.007 | 4.595 | | 5.158 | 5.844 | 4.632 | | 4.973 | 4.919 |  |  |
| 60 | 4.916 | | | 3.99 | 4.454 | | 5.147 | 5.712 | 5.986 | | 4.385 | 5.766 |  |  |
|  |  | | |  |  | |  |  |  | |  |  |  |  |
| Time (min) | Sage (400 mg/kg/day.bid) | | | | | | | | | | | |  |  |
| -10 | 7.562 | | | 5.271 | 7.227 | | 6.686 | 5.012 | 6.165 | | 7.738 | 5.993 |  |  |
| 0 | 9.003 | | | 6.775 | 7.849 | | 8.158 | 7.332 | 7.954 | | 8.839 | 7.461 |  |  |
| 10 | 7.293 | | | 6.832 | 6.947 | | 8.448 | 7.652 | 7.942 | | 9.234 | 8.393 |  |  |
| 20 | 9.621 | | | 5.06 | 5.599 | | 6.902 | 4.724 | 6.203 | | 6.952 | 6.64 |  |  |
| 30 | 8.875 | | | 4.635 | 5.456 | | 6.999 | 4.624 | 5.927 | | 5.723 | 5.557 |  |  |
| 45 | 8.67 | | | 4.991 | 6.218 | | 6.62 | 4.325 | 6.168 | | 6.737 | 5.192 |  |  |
| 60 | 8.378 | | | 4.459 | 5.713 | | 6.286 | 4.323 | 6.733 | | 6.874 | 4.864 |  |  |
|  |  | | |  |  | |  |  |  | |  |  |  |  |
|  |  | | |  |  | |  |  |  | |  |  |  |  |
| Time (min) | Rosi.(3mg/kg/day.bid) | | | | | | | | | | | |  |  |
| -10 | 5.338 | | | 5.107 | 4.662 | | 5.484 | 4.384 | 5.86 | | 5.711 | 5.46 |  |  |
| 0 | 7 | | | 7.631 | 7.731 | | 6.905 | 6.633 | 7.17 | | 7.59 | 7.332 |  |  |
| 10 | 6.859 | | | 6.477 | 7.825 | | 6.156 | 6.525 | 7.412 | | 7.652 | 8.517 |  |  |
| 20 | 4.925 | | | 4.588 | 4.618 | | 6.054 | 4.081 | 5.163 | | 6.29 | 5.931 |  |  |
| 30 | 4.202 | | | 4.809 | 4.169 | | 6.425 | 3.761 | 4.482 | | 4.585 | 6.082 |  |  |
| 45 | 3.969 | | | 4.876 | 4.454 | | 5.036 | 3.714 | 5.086 | | 4.979 | 6.163 |  |  |
| 60 | 3.468 | | | 4.578 | 3.837 | | 3.891 | 2.99 | 4.723 | | 4.522 | 4.737 |  |  |
|  |  | | |  |  | |  |  |  | |  |  |  |  |

**Raw Data for Figure 4**

**Figure 4A**

| vehicle | Sage (100 mg/kg/day.bid) | Sage (400 mg/kg/day.bid) | Rosi.(3mg/kg/day.bid) |
| --- | --- | --- | --- |
|  | 1458.1550 | 1563.8330 | 371.3767 |
| 4564.7250 | 678.7983 | 719.0850 | 1810.8550 |
| 856.6300 | 1147.8520 | 1100.4300 | 479.1383 |
| 540.4400 | 536.9383 | 1558.5550 | 514.1500 |
| 3680.2820 | 713.8300 | 1874.4750 | 117.6050 |
| 4165.7550 | 1103.9620 | 948.6733 | 407.3167 |
| 2241.1320 | 772.5383 | 1040.7600 | 695.4383 |
| 573.7150 | 1212.8220 | 825.9600 | 540.4400 |

**Figure 4B**

| vehicle | Sage (100 mg/kg/day.bid) | Sage (400 mg/kg/day.bid) | Rosi.(3mg/kg/day.bid) |
| --- | --- | --- | --- |
|  | 24.7440 | 19.4080 | 21.6480 |
| 6.5320 | 7.2040 | 22.1240 | 29.4520 |
| 29.8320 | 16.6880 | 9.4200 | 23.5840 |
| 15.9720 | 25.8200 | 30.2320 | 14.1320 |
| 30.3760 | 12.7400 | 21.3240 | 4.8000 |
| 3.4960 | 28.6920 | 27.9600 | 20.5360 |
| 29.4840 | 20.1760 | 29.3480 | 8.7360 |
| 20.2560 | 27.8680 | 20.3680 | 3.8280 |

**Figure 4C**

| vehicle | Sage (100 mg/kg/day.bid) | Sage (400 mg/kg/day.bid) | Rosi.(3mg/kg/day.bid) |
| --- | --- | --- | --- |
|  | 5.988 | 6.161 | 7.217 |
| 7.406 | 4.758 | 5.972 | 6.697 |
| 4.065 | 6.240 | 5.736 | 6.729 |
| 5.373 | 6.209 | 5.452 | 5.862 |
| 6.792 | 5.878 | 6.335 | 2.315 |
| 6.508 | 6.177 | 5.405 | 5.468 |
| 6.697 | 5.231 | 6.146 | 6.981 |
| 5.074 | 5.420 | 5.862 | 7.454 |

**Figure 4D**

| vehicle | Sage (100 mg/kg/day.bid) | Sage (400 mg/kg/day.bid) | Rosi.(3mg/kg/day.bid) |
| --- | --- | --- | --- |
|  | 2.723 | 3.519 | 2.641 |
| 2.833 | 2.641 | 2.195 | 3.918 |
| 2.483 | 3.313 | 3.304 | 3.145 |
| 2.536 | 3.131 | 3.572 | 2.195 |
| 4.033 | 3.049 | 3.659 | 1.379 |
| 2.728 | 3.356 | 3.476 | 2.310 |
| 3.232 | 2.574 | 3.759 | 3.625 |
| 2.622 | 2.709 | 2.430 | 2.996 |

**Figure 4E**

| vehicle | Sage (100 mg/kg/day.bid) | Sage (400 mg/kg/day.bid) | Rosi.(3mg/kg/day.bid) |
| --- | --- | --- | --- |
|  | 1.458 | 1.327 | 1.779 |
| 2.109 | 0.882 | 1.812 | 1.302 |
| 1.113 | 0.895 | 1.862 | 1.228 |
| 1.911 | 0.866 | 1.392 | 1.384 |
| 1.689 | 0.964 | 2.380 | 0.841 |
| 1.524 | 1.360 | 1.779 | 1.137 |
| 1.343 | 0.931 | 0.857 | 1.014 |
| 1.705 | 1.252 | 2.528 | 0.726 |

**Figure 4F**

| vehicle | Sage (100 mg/kg/day.bid) | Sage (400 mg/kg/day.bid) | Rosi.(3mg/kg/day.bid) |
| --- | --- | --- | --- |
|  | 0.6090 | 0.7225 | 1.0060 |
| 1.0260 | 0.4165 | 0.9985 | 0.6170 |
| 0.5065 | 0.4765 | 0.5990 | 0.3835 |
| 0.9180 | 0.3985 | 0.6095 | 0.4715 |
| 0.8150 | 0.7075 | 0.8985 | 0.8705 |
| 0.5340 | 0.8000 | 0.8830 | 0.6370 |
| 0.8250 | 0.5190 | 0.4990 | 0.3830 |
| 0.6850 | 0.6745 | 0.8050 | 0.5040 |

**Raw Data for Figure 5**

**Figure 5A**

|  | Vehicle | | | | | | | |
| --- | --- | --- | --- | --- | --- | --- | --- | --- |
| IL-10 |  | 18.917 | 2.502 | 35.273 | 65.119 | 34.931 | 47.550 | 20.533 |
| IL-5 |  | 35.046 | 17.725 | 17.008 | 33.310 | 20.643 | 20.540 | 20.550 |
| IL-4 |  | 0.944 | 3.480 | 0.456 | 2.852 | 0.893 | 1.093 | 1.583 |
| IL-2 |  | 2.003 | 2.410 | 3.409 | 5.985 | 0.653 | 1.994 | 0.623 |
|  |  |  |  |  |  |  |  |  |
|  | Sage (100 mg/kg/day.bid) | | | | | | | |
| IL-10 | 78.668 | 60.065 | 83.998 | 65.593 | 38.629 | 48.601 | 58.644 | 58.696 |
| IL-5 | 12.424 | 28.150 | 15.977 | 14.252 | 12.898 | 23.533 | 13.213 | 21.354 |
| IL-4 | 1.204 | 6.245 | 5.264 | 1.539 | 3.553 | 3.378 | 3.451 | 10.234 |
| IL-2 | 11.341 | 6.418 | 7.744 | 10.086 | 8.202 | 3.600 | 11.319 | 1.994 |

**Figure 5B**

|  | Vehicle | | | | | | | |
| --- | --- | --- | --- | --- | --- | --- | --- | --- |
| IFNγ |  | 2.467 | 1.418 | 4.798 | 0.997 | 2.807 | 2.828 | 2.236 |
| TNFα |  | 59.211 | 23.352 | 52.653 | 40.166 | 26.415 | 39.112 | 31.161 |
| KC/GRO |  | 260.916 | 147.442 | 216.962 | 322.551 | 269.478 | 383.925 | 362.720 |
| IL-6 |  | 79.353 | 21.921 | 49.645 | 63.270 | 102.682 | 29.314 | 105.363 |
| IL-12 |  | 197.718 | 169.319 | 232.330 | 195.747 | 147.295 | 72.542 | 190.483 |
| IL-1β |  | 1.563 | 2.391 | 0.962 | 2.116 | 1.778 | 4.248 | 4.648 |
|  |  |  |  |  |  |  |  |  |
|  |  |  |  |  |  |  |  |  |
|  | Sage (100 mg/kg/day.bid) | | | | | | | |
| IFNγ | 0.586 | 2.129 | 0.997 | 2.053 | 4.117 | 1.661 | 3.719 | 4.375 |
| TNFα | 25.198 | 18.973 | 24.476 | 32.441 | 19.035 | 40.517 | 24.126 | 19.406 |
| KC/GRO | 150.872 | 128.789 | 170.576 | 265.911 | 112.329 | 202.847 | 115.215 | 119.194 |
| IL-6 | 6.754 | 34.225 | 28.741 | 71.937 | 33.656 | 46.293 | 112.422 | 86.408 |
| IL-12 | 86.560 | 183.175 | 81.673 | 73.952 | 67.254 | 129.100 | 92.814 | 181.906 |
| IL-1β | 2.116 | 1.078 | 1.793 | 1.012 | 1.799 | 3.030 | 0.180 | 2.391 |
